# Evaluation Exploration of Atlas-Based and Deep Learning-Based Automatic Contouring for Nasopharyngeal Carcinoma

**DOI:** 10.3389/fonc.2022.833816

**Published:** 2022-03-31

**Authors:** Jinyuan Wang, Zhaocai Chen, Cungeng Yang, Baolin Qu, Lin Ma, Wenjun Fan, Qichao Zhou, Qingzeng Zheng, Shouping Xu

**Affiliations:** ^1^ Department of Radiation Oncology, The First Medical Center of the Chinese PLA General Hospital, Beijing, China; ^2^ Manteia Technologies Co., Ltd., Xiamen, China; ^3^ Department of Radiation Oncology, Beijing Geriatric Hospital, Beijing, China

**Keywords:** atlas, deep learning (DL), training, nasopharyngeal carcinoma (NPC), auto-segmentation, organs at risk (OARs)

## Abstract

**Purpose:**

The purpose of this study was to evaluate and explore the difference between an atlas-based and deep learning (DL)-based auto-segmentation scheme for organs at risk (OARs) of nasopharyngeal carcinoma cases to provide valuable help for clinical practice.

**Methods:**

120 nasopharyngeal carcinoma cases were established in the MIM Maestro (atlas) database and trained by a DL-based model (AccuContour^®^), and another 20 nasopharyngeal carcinoma cases were randomly selected outside the atlas database. The experienced physicians contoured 14 OARs from 20 patients based on the published consensus guidelines, and these were defined as the reference volumes (V_ref_). Meanwhile, these OARs were auto-contoured using an atlas-based model, a pre-built DL-based model, and an on-site trained DL-based model. These volumes were named V_atlas_, V_DL-pre-built_, and V_DL-trained_, respectively. The similarities between V_atlas_, V_DL-pre-built_, V_DL-trained_, and V_ref_ were assessed using the Dice similarity coefficient (DSC), Jaccard coefficient (JAC), maximum Hausdorff distance (HD_max_), and deviation of centroid (DC) methods. A one-way ANOVA test was carried out to show the differences (between each two of them).

**Results:**

The results of the three methods were almost similar for the brainstem and eyes. For inner ears and temporomandibular joints, the results of the pre-built DL-based model are the worst, as well as the results of atlas-based auto-segmentation for the lens. For the segmentation of optic nerves, the trained DL-based model shows the best performance (p < 0.05). For the contouring of the oral cavity, the DSC value of V_DL-pre-built_ is the smallest, and V_DL-trained_ is the most significant (p < 0.05). For the parotid glands, the DSC of V_atlas_ is the minimum (about 0.80 or so), and V_DL-pre-built_ and V_DL-trained_ are slightly larger (about 0.82 or so). In addition to the oral cavity, parotid glands, and the brainstem, the maximum Hausdorff distances of the other organs are below 0.5 cm using the trained DL-based segmentation model. The trained DL-based segmentation method behaves well in the contouring of all the organs that the maximum average deviation of the centroid is no more than 0.3 cm.

**Conclusion:**

The trained DL-based segmentation performs significantly better than atlas-based segmentation for nasopharyngeal carcinoma, especially for the OARs with small volumes. Although some delineation results still need further modification, auto-segmentation methods improve the work efficiency and provide a level of help for clinical work.

## Introduction

Nasopharyngeal carcinoma (NPC) is a common malignant tumor in the head and neck region. Its annual incidence rate is about 25 to 30 cases per 100,000 people, and this rate is increasing year by year (1, 2). Most patients with NPC will undergo radiotherapy. Radiation delivers the target area, inevitably exposing normal tissue around the target. The incidence of adverse symptoms occurs directly related to the dose received by organs at risk (OARs) (3, 4). Since eliminating the unnecessary irradiation dose of organs can reduce the side effects and improve life quality, precise radiation therapy (RT) becomes particularly important (5). The rapid development of new technology has continuously improved the accuracy of RT. Nonetheless, the precise delineation of tumors and OARs before RT is essential. Therefore, we can quantitatively evaluate OARs and thus ensure proper treatment after dose calculation.

Due to the lack of contrast to CT images, the indistinct boundaries, and the numerous organs, physicians need to delineate the targets and OARs manually for NPC. This process becomes cumbersome; it also requires time and workforce, resulting in a relatively low efficiency (6, 7). To simplify the heavy task of contouring, many software tools for automatic delineation have appeared from the market, most of which use atlas-based auto-segmentation (ABAS) methods (8–11), simultaneously, with the application of machine learning technique and intense learning methods in RT (12–14). These artificial neural network-based methods may offer better performance. Nevertheless, manual delineation is still the standard procedure for most medical institutions.

This study intends to compare the results from an atlas-based and deep learning (DL)-based segmentation method of organs at risk (OARs) for NPC to evaluate the difference between the two methods and explore conclusions for clinical practice.

## Materials and Methods

### Data Source and Study Design

This retrospective study included a total of 140 patients with NPC who were treated at our institution from July 2016 to July 2018. All the CT image data we selected were acquired in supine position with a thermoplastic head and neck mask for each patient, using a Siemens SOMATOM Definition AS CT scanner with a selected slice thickness of 3 mm, a valid mAs of 300, a tube voltage of 120 kV, and a matrix of 512 × 512. We excluded the cases that were too fat or too slim because the head and neck region was not so big as other parts of the body, such as the abdomen; there were not so many cases that did not meet. After scanning, we loaded the CT images into TPS (Pinnacle, Version 10.0). According to the published consensus guidelines and the fusion with MR or contrast CT, all the OAR delineations were done manually by the same experienced clinician, defined as V_ref_ (V_ref_, the reference volume). In addition, the study randomly selected 120 CT images and their structure files then transferred them into MIM Maestro software (Version 6.6.5) to establish the atlas library, and we used the same 120 CT images and their structure files to train the DL-based model (AccuLearning^®^, a Commercial Company). Also, the pre-built automatic segmentation model (AccuContour^®^, a Commercial Company) is used for comparison. Finally, we used the atlas-based, pre-built, and on-site trained DL-based automatic segmentation methods to automatically delineate the remaining 20 patients and define them as V_atlas_, V_DL-pre-built_, and V_DL-trained_, respectively. This study selected 14 OARs, including brainstem, eyes, lens, optic nerves, inner ears, temporomandibular joints (TMJs), parotid glands, and oral cavity.

### Automatic Segmentation Methods

Atlas-based auto-segmentation used CT images with current delineation data as template images to build an atlas database, compared the new CT images with the others in the databases, then found the best match. Moreover, the new CT images were registered to the template CT images using intensity-based deformable registration. We transformed the contours of the template CT into the new CT image through the deformation registration parameters and obtained the final contouring result. Our study chose a uniform case as the atlas template CT (reference CT). The new cases were added as a subset into the atlas library after the re-registration with the template CT. Moreover, we used the majority vote algorithm and match number 3 to do the atlas auto-segmentation using MIM Maestro software.

The network model structure in our study is residue-Unet, with the loss function of Dice function integrated into AccuContour produced by a commercialized product. Both encoder and decoder are composed of 5 cascades of Residual blocks, and each Residual block is composed of two convolution layers with the convolution kernel size of 3 × 3. Each residual block is cascaded with the downsampling and upsampling layers. The downsampling method is maximum pooling, and the upsampling method is a nearest-neighbor interpolation. The pre-built auto-segmentation model was based on thousands of finely labeled cases from various medical centers except our institution. The on-site trained auto-segmentation model was based on the cases from our institution. According to information disclosed to the public, the developer utilized standard methods including supervised or semi-supervised learning, prior knowledge aggregation, and ROI-specific post-processing to obtain the pre-built model. The company also provides a high-performance DL research platform called AccuLearning for user-customizable on-site training needs. Preprocessing standardizes and resamples images using adaptive rules based on image intensity range and distribution characteristics. Data augmentation is first conducted on the loaded images, and then balanced cropping is performed accordingly to the label area to generate the model input. Model training utilizes an adaptive network structure adjusting strategy based on gradient feedback and the loss function’s progress to accommodate the training dataset’s characteristics.

### Quantitative Evaluation

We evaluated the results using four parameters, including Dice similarity coefficient (DSC), Jaccard coefficient (JAC), maximum Hausdorff distance (HD_max_), and deviation of the centroid (DC). One RT physicist performed all atlas and DL delineation tasks to prevent any possible differences among the operators.

1. Dice similarity coefficient (DSC) (15)

The DSC is defined to be the ratio of the intersection to the average area.


$$\text{DSC}=\frac{2\cdot (V_{ref}\cap V_{auto})}{V_{ref}+V_{auto}}$$

where V_ref_ is the reference delineation volume and V_auto_ is the auto-segmentation volume.

2 Jaccard index (JAC) (16)

The JAC is defined to be the ratio of the intersection to the union area.


$$\text{JAC}=\frac{V_{ref}\cap V_{auto}}{V_{ref}\cup V_{auto}}$$

where V_ref_ is the reference delineation volume and V_auto_ is the auto-segmentation volume.

3 Maximum Hausdorff distance (HD_max_) (17)

Suppose there are two groups of sets X = {x_1_, …, x_n_}, Y={y_1_, …, y_n_}, then the maximum Hausdorff distance between these two sets of points is defined as


HD(X,Y)=max(h(X,Y),h(Y,X))

where $h\left(X,Y\right)=\underset{x\in X}{max}\underset{y\in Y}{min}\left\|x-y\right\|$

4 Deviation of centroid (DC) (18)

The DC is defined as the deviation of centroid of two volumes.


$$\text{DC}=\sqrt{{\left(x_{auto}-x_{ref}\right)}^{2}+{\left(y_{auto}-y_{ref}\right)}^{2}+{\left(z_{auto}-z_{ref}\right)}^{2}}$$

### Statistical Analysis Methods

The one-way ANOVA test (SPSS, Version 23; SPSS Inc, Chicago, USA) was applied to measure the difference in evaluation parameters with an LSD (least significance difference) method to do the *post hoc* multiple comparisons (V_atlas_ vs. V_DL-pre-built_ vs. V_DL-trained_). A *p*-value less than 0.05 (typically ≤0.05) was statistically significant.

## Results

**Figure 1:**
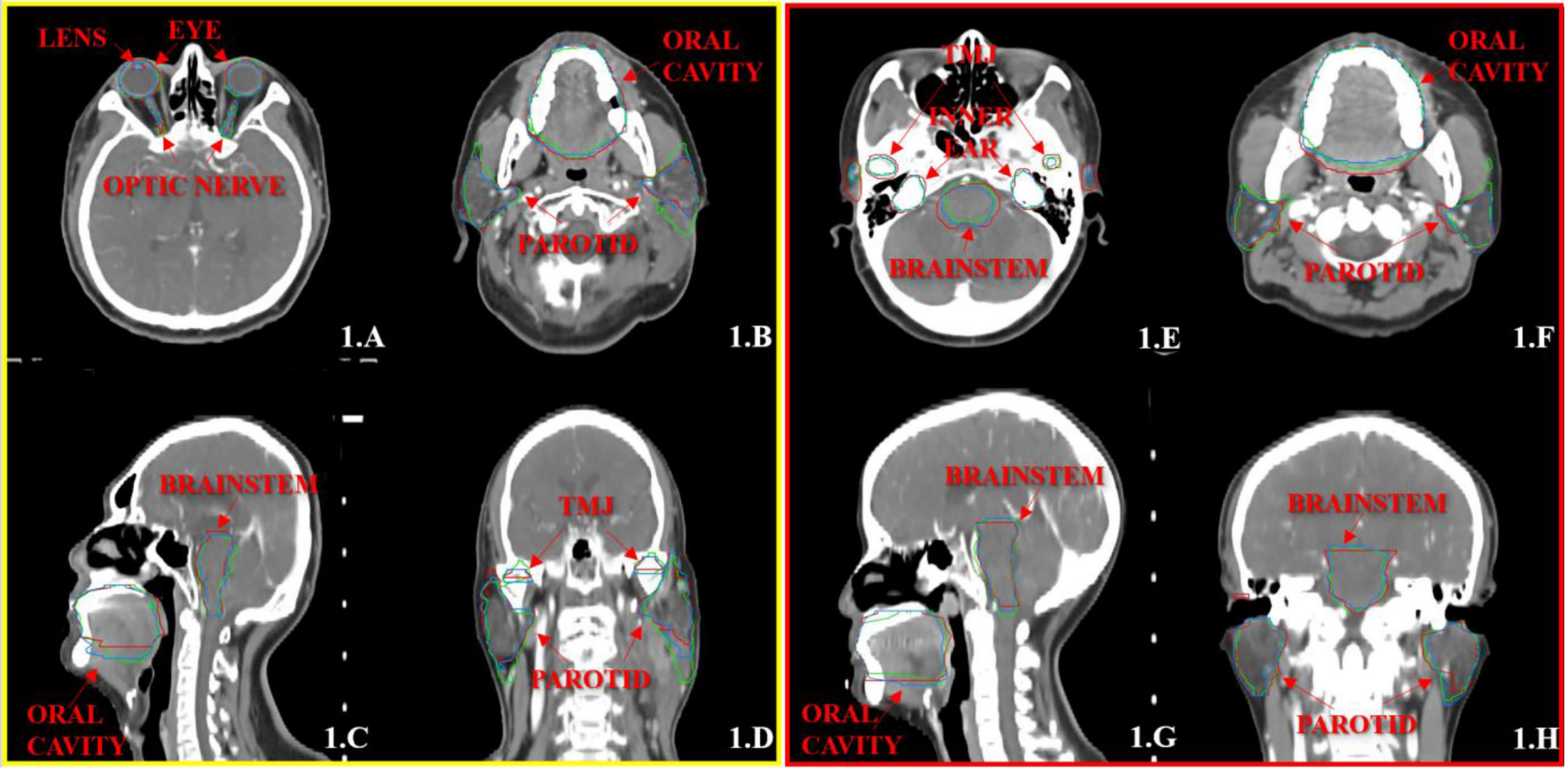
The segmentation results on the atlas and trained DL-based models for two representative cases with the transverse **(A, B, E, F)**, sagittal **(C, G)**, and coronal **(D, H)** images, respectively. The ground-truth delineations are depicted in red, the automatic delineations based on the atlas model are depicted in green, and the automatic delineations based on the trained DL model are depicted in blue. Case 1 is shown in **(A–D)**, and case 2 is shown in **(E–H)**.

**Figure 2:**
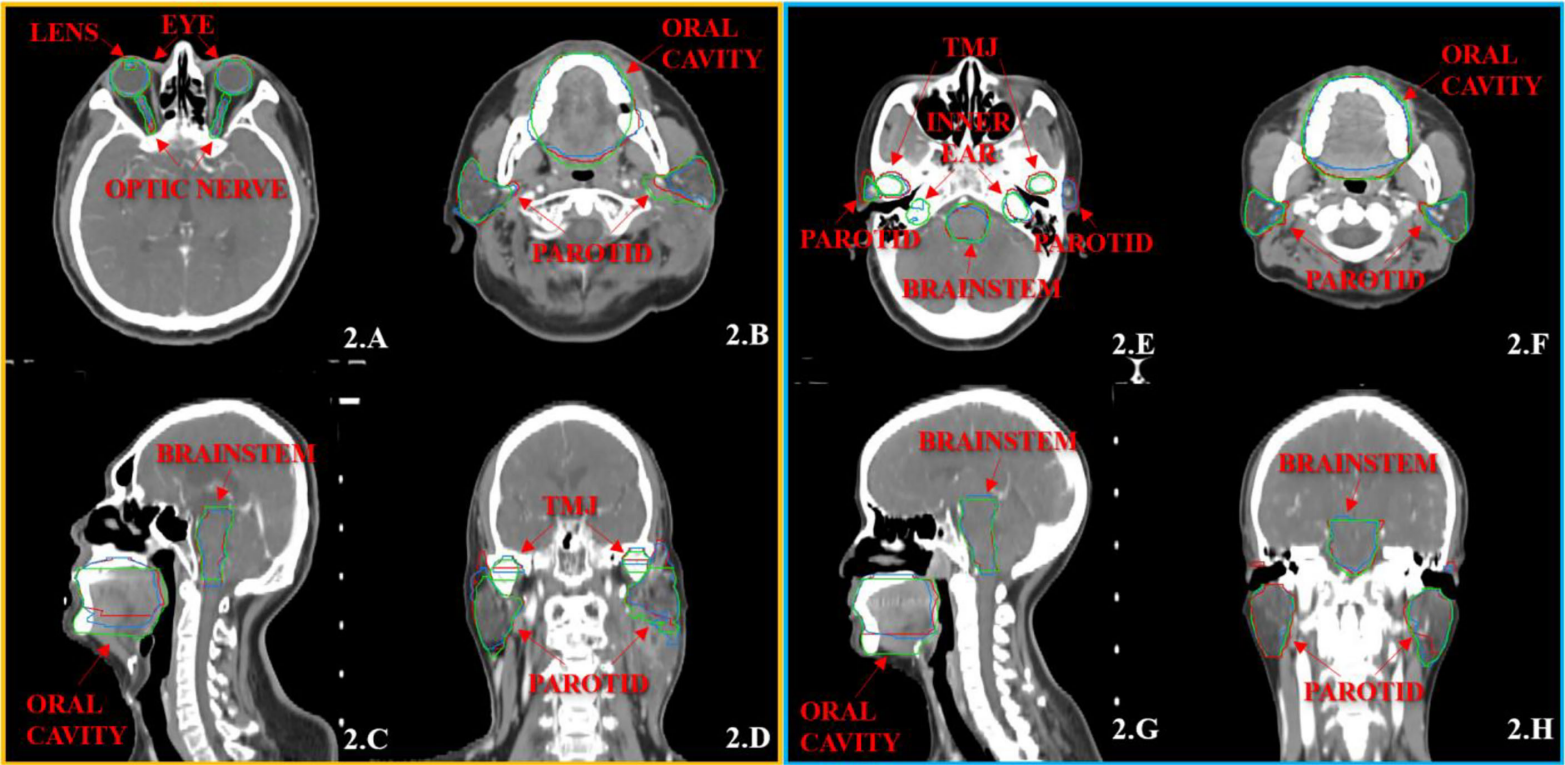
The segmentation results on the pre-built and trained DL-based models for the two same cases with the transverse **(A, B, E, F)**, sagittal **(C, G)**, and coronal **(D, H)** images, respectively. The ground-truth delineations are depicted in red, the automatic delineations based on the pre-built DL-based model are depicted in green, and the automatic delineations based on the trained DL-based model are depicted in blue. Case 1 is shown in **(A–D)**, and case 2 is shown in **(E–H)**.

**Figure 3:**
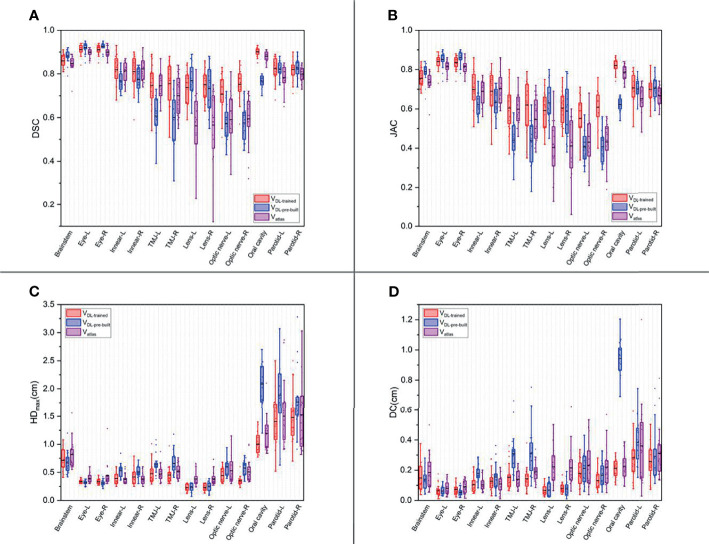
The box plots of four quantitative evaluation parameters of the OARs for the three segmentation methods. The results of DSC are listed in **(A)**, JAC in **(B)**, HD_max_ in **(C)**, and DC in **(D)**. The results of the trained DL-based model are depicted in red, the pre-built DL-based model in blue, and the atlas-based auto-segmentation in purple.

**Figures 1**, **2** show the contouring results of two real clinical test cases of the three methods, respectively, where **Figures 1A–D**, **2A–D** show an identical case, and **Figures 1E–H**, **2E–H** show the same case similarly. To an intuitive evaluation of the contouring accuracy, the results of four quantitative evaluation parameters of the OARs for the three segmentation methods, including DSC, JAC, HD_max_, and DC, are presented in the form of box plots (**Figure 3**), and the statistical analysis results of the four parameters are summarized in **Table 1**.

**Table 1:** The statistical analysis results of the four quantitative evaluation parameters for three segmentation methods.

	Brainstem	Eye-L	Eye-R	Inner ear-L	Inner ear-R	TMJ-L	TMJ-R
DSC	0.002*^ac^*	0.015*^c^*	0*^ac^*	0.004*^ac^*	0.052*^c^*	0*^ac^*	0*^ac^*
JAC	0.002*^ac^*	0.009*^bc^*	0*^ac^*	0.003*^ac^*	0.041*^c^*	0*^ac^*	0*^ac^*
HD_max_	0.071*^c^*	0*^bc^*	0.004*^bc^*	0*^ac^*	0.006*^ac^*	0.003*^ac^*	0.001*^ac^*
DC	0.252	0.360	0.007*^c^*	0*^ac^*	0.123*^c^*	0*^ac^*	0*^ac^*
	**Lens-L**	**Lens-R**	**Optic nerve-L**	**Optic nerve-R**	**Oral Cavity**	**Parotid-L**	**Parotid-R**
DSC	0*^bc^*	0*^bc^*	0*^ab^*	0*^ab^*	0*^abc^*	0.018*^bc^*	0.100*^c^*
JAC	0*^bc^*	0*^bc^*	0*^ab^*	0*^ab^*	0*^abc^*	0.019*^bc^*	0.099*^c^*
HD_max_	0*^bc^*	0*^bc^*	0.047*^a^*	0*^ab^*	0*^ac^*	0.025*^ac^*	0.210
DC	0*^bc^*	0*^bc^*	0.348	0.019*^b^*	0*^ac^*	0.220	0.522

Each symbol represents significant differences between different groups (p < 0.05).

^a^Indicates a significant difference between V_DL-trained_ and V_DL-pre-built_.

^b^Indicates a significant difference between V_DL-trained_ and V_atlas_.

^c^Indicates a significant difference between V_DL-pre-built_ and V_atlas_.

For DSC results, although the results of V_DL-pre-built_ were better than the other two in the contouring of brainstem and eyes (*p* < 0.05), all of the DSC values of the three were at a relatively high level (the DSC of the brainstem was more than 0.85, and the DSC values of eyes were floating around 0.9). For inner ears and TMJs, the pre-built DL-based model results were worse than the other two, with no significant differences between the two. Moreover, for the lens, the values of atlas-based auto-segmentation were the worst (*p* < 0.05), and there were no significant differences between the trained and pre-built DL-based models results. For the optic nerves, the trained DL-based model showed the best performance. For the contouring of the oral cavity, the DSC value of V_DL-pre-built_ was the worst, and that of the V_DL-trained_ was the best (*p* < 0.05). Then for the parotid glands, the DSC value of V_atlas_ was the minimum (about 0.80 or so), and those of V_DL-pre-built_ and V_DL-trained_ were slightly larger (about 0.82 or so). The JAC results were almost the same as the volume-related parameter of DSC.

For HD_max_, the atlas-based auto-segmentation showed relatively large values in delineating the brainstem, eyes, lens, and optic nerves. The pre-built DL-based model method showed a lousy performance of delineation in the inner ears, TMJs, optic nerves, oral cavity, and parotid glands. For the brainstem, eyes, and lens, the HD_max_ of the atlas-based method was much larger than that of DL-based methods. In addition to the oral cavity, parotid glands, and the brainstem, the HD_max_ values of the other organs were all below 0.5 cm using the trained DL segmentation model.

V_atlas_ showed more significant results for the DC value in the brainstem, eyes, lens, and optic nerves, and the V_DL-pre-built_ showed larger values in the inner ears, TMJs, optic nerves, and oral cavity. The trained DL-based segmentation method performed well in the contouring of all the organs that the maximum average DC is no more than 0.3 cm.

## Discussion

Although it is time-consuming and intra-observer and inter-observer differences usually occur, scholars worldwide have been trying to find a more rapid and more accurate method or evaluate the already existing processes. Scholars have recently published many DL studies on auto-segmentation (19–23), involving various algorithms and machine learning techniques, especially DL methods. Yang et al. (24) evaluated a U-net-based whole convolutional neural network (CNN). They got the conclusion that DL-based auto-segmentation showed great potential to alleviate the labor-intensive contouring of OARs for RT treatment planning. These methods based on artificial neural networks, especially after retraining, have shown excellent functionality better than most classification and regression methods. This conclusion is consistent with our study.

The clinical applicability of atlas-based auto-segmentation has been reported many times in the head and neck, chest, abdomen, and pelvic diseases (25–28). The results show that the atlas-based auto-segmentation outcomes could meet the clinical application and significantly reduce manual labor. Dijk et al. (29) evaluated two image segmentation methods, atlas-based segmentation and convolutional neural network-based DL model segmentation. They collected the contours of 589 head and neck cancer patients from clinical practice and used them to train models. DL-based segmentation showed encouraging results compared to the ABAS. The same is true for the findings of our research.

The time spent on contouring roughly depends on two main factors, the visualization of organ boundaries and the volume of OARs. For software and human observers, high-contrast edges are easier to detect; otherwise, low-contrast borders are more challenging to notice. The automatic segmentation method is generally inaccurate for the boundaries of minor soft tissue, which increases the time spent on adjustment. Most importantly, it is also difficult for observers to distinguish the boundaries. These increase the time required for adjustment additionally. Even if we assume that automatic segmentation techniques will achieve human-level performance for contouring in the future, human observers may still need to evaluate the contouring results for some difficult situations.

The accurate and reliable segmentation of NPC images is essential in clinical applications (including RT). However, the targets of NPC vary in size and shape, as well as variable intensity within the tumor and similar intensity to nearby tissues, which makes the segmentation task more difficult. The emergence of automatic segmentation software has provided convenience for RT undoubtedly, especially for adaptive radiotherapy, increases the efficiency of delineation, and reduces the variety in contouring to a certain extent. However, we could not ignore the influence of software differences on the delineation outcomes.

In our study, the atlas-based, pre-built, and trained DL-based automatic segmentation methods had good performances for the segmentation of the brainstem and eyes. The three methods got good results of DSC (e.g., the DSC results of the brainstem were above 0.8, the results of the deviation of the centroid were below 0.2 cm, the DSC results of the eyes were above 0.9, and the results of the deviation of centroid were below 0.1 cm). The reasons were that the position of the eyes was relatively fixed and the boundaries relatively straightforward, and all three methods could make better identification. The boundary of the brainstem was not clear, but the position was fixed, so we could still get a good result.

For the inner ears, TMJs, optic nerves, and oral cavity, the trained DL-based model showed a vast improvement toward the pre-built model (**Figure 2**) because these contours were more subjectively affected by physicians. Each institution or even observer might have a different contouring habit for organs. It is not easy to achieve an overall contouring agreement so that the specificity of these organs is relatively high. So when one wants to use a DL-based method for the auto-segmentation of the OARs, they need to use their data to train the model, or even the same physician’s patients, to achieve satisfactory results. Atlas-based segmentation performed a relatively poor performance of the lens and optic nerves. One possible reason might be that the volume of these organs was much smaller (24). The atlas-based method was at a disadvantage of segmenting small volume organs than the DL method.

Another problematic point is the delineation of the parotid glands because of the unclear boundary and the variable contouring habits of each physician. Our study showed that the contouring results of parotid glands with the atlas-based method include most of the central areas of parotid glands, and there was still a lack in the contouring accuracy of the boundary. Meanwhile, the trained DL-based model slightly increased in the volume of parotid glands, and the boundary was much closer to the ground truth (**Figure 1**).

The contouring time of these methods in our research was not listed in the quantitative evaluation because the contouring time was so fast, about 3 min for each patient through estimation that was far less than the manual delineation time. Still, the time to build an atlas database was cumbersome. Establishing an atlas database required manual handling of each patient, which spent about 3 h for 120 patients. At the same time, the training time of the DL model was just about 49 min, with no need for a large amount of human intervention. We only chose 120 patients for the atlas database and training model in this study. The DL model for training will have a significant advantage if more data models are added in the future, avoiding the cumbersome establishment cost and the choice of an individual situation.

At the beginning of our study, we compared the delineation of the pre-built DL- and atlas-based method and added the on-site trained model later. It is, this step that made the contouring result in a significant improvement of both volume and distance (for example, the DSC values of the inner ears were increased by 0.05 and HD_max_ decreased by 1 mm, the DSC values of TMJs were increased by about 0.15 and HD_max_ decreased by 1.9 mm, the DSC values of the optic nerves were increased by about 0.16 and HD_max_ decreased by 2 mm, the DSC values of the oral cavity were increased by 0.13 and HD_max_ decreased by 16 mm, and the centroid distance decreased by 7 mm and the HD_max_ of the parotid glands were decreased by 4 mm).

In this study, the trained DL-based model performed brilliantly, showing a good result in the contouring of each organ, and all the mean DSC values were more than 0.7 (**Figure 3A**) (30), which met the clinical standards. In particular, the widely accepted optic nerves, because of the small size and unclear boundaries, have a relatively low accuracy of DSC values in many studies (14, 31–33). Similarly, the optic nerves had worse accuracy than other organs in our results. Still, the mean DSC values were above 0.7. We analyzed the excellent performance as a result of model selection. Although some studies demonstrate that a better performance needs diversity and numerous training datasets, all the cases used in our study are contoured only by one experienced oncologist. Compared to the open-access resource platform of medical images for cancer research, our datasets were more specific, eliminating the intra- and inter-observer variability to the utmost extent with very high quality and representativeness, which made the trained model more characteristic to meet the clinical acceptance. Lin et al. (34) summarized the major deep learning architectures related to target volume segmentation, surveyed the use of three common imaging modalities (CT, MRI, PET) in radiation therapy, and compared their performance. They pointed out that high-quality annotated data were a big challenge for deep learning models, and deep learning-based automatic segmentation had great potential.

It is worth mentioning that the atlas-based method also showed a good ability for the segmentation of OARs of NPC. Still, to ensure a better result, one needs to use their contouring cases to build the atlas database, especially for some specific organs. In institutions with no use conditions of DL segmentation models, the application of the atlas-based method could still meet the clinical demands for most organs. Our research innovation combined the atlas-based, pre-built, and on-site trained DL-based automatic segmentation methods, providing intuitive results for clinical applications.

A limitation of this study is that we only used 120 cases to train the DL model to maintain the consistency of the atlas library. Perhaps with more data added to the DL model, the delineation results will be further improved, and we also have reasons to believe this. The diversity of training data and the automatic segmentation of other organs or targets for the whole body are also the focus of our following study. With the rapid development of multi-imaging modalities (such as CT, MR, PET) and radiotherapy technology (such as MRI-Linac), deep learning-based automatic segmentation methods have more vast fields.

## Conclusions

Although some delineation outcomes still need further modification, the trained DL-based auto-segmentation method performs better than the atlas-based segmentation method that benefits clinical efficiency, optimizes the treatment procedure, and provides a certain level of help for clinical work. For clinical applications, the DL-based automatic segmentation of OARs can significantly save time for physicians. More factors that influence the accuracy of automatic segmentation in clinical applications still need further exploration.

## Data Availability Statement

The original contributions presented in the study are included in the article/supplementary material. Further inquiries can be directed to the corresponding authors.

## Author Contributions

JW: experiment design and article writing. ZC, CY, and QZhou: technical support. BQ and LM: data quality control. WF and QZheng: data collection. SX: conception, experiment design, and article modification. All authors contributed to the article and approved the submitted version.

## Funding

This work was supported by the National Natural Science Foundation of Chinese Science Foundation Project (81801799)

## Conflict of Interest

Authors ZC, CY, and QZheng were employed by Manteia Technologies Co., Ltd.

The remaining authors declare that the research was conducted in the absence of any commercial or financial relationships that could be construed as a potential conflict of interest.

## Publisher’s Note

All claims expressed in this article are solely those of the authors and do not necessarily represent those of their affiliated organizations, or those of the publisher, the editors and the reviewers. Any product that may be evaluated in this article, or claim that may be made by its manufacturer, is not guaranteed or endorsed by the publisher.
